# Growth of *Methylobacterium organophilum* in Methanol for the Simultaneous Production of Single-Cell Protein and Metabolites of Interest

**DOI:** 10.17113/ftb.60.03.22.7372

**Published:** 2022-09

**Authors:** Ana Cristina Pantoja Simões, Rodrigo Pimentel Fernandes, Maysa Silva Barreto, Gabriela Bouça Marques da Costa, Mateus Gomes de Godoy, Denise Maria Guimarães Freire, Nei Pereira

**Affiliations:** 1 Laboratories of Bioprocess Development, School of Chemistry, Federal University of Rio de Janeiro, RJ 21941-972, Brazil; 2 Microbial Biotechnology Laboratory, Institute of Chemistry, Exact and Natural Sciences Center of the Federal University of Rio de Janeiro, RJ 21941-972, Brazil; 3 Laboratory of Biotechnology and Microbial Ecology, Institute of Microbiology, Federal University of Rio de Janeiro, RJ 21941-902, Brazil

**Keywords:** single cell protein, methylotrophic bacterium, methanol consumption

## Abstract

**Research background:**

This study aims to monitor the growth of the methylotrophic bacteria *Methylobacterium organophilum* in a culture medium with methanol as a carbon source and to verify the production of unicellular proteins and other biomolecules, such as carotenoids, exopolysaccharides and polyhydroxyalkanoates, making them more attractive as animal feed.

**Experimental approach:**

Bacterial growth was studied in shake flasks using different carbon/nitrogen (C:N) ratios to determine their best ratio for achieving the highest volumetric productivity of cells and substrate consumption rate. This optimal parameter was further used in a fed-batch operating bioreactor system to define the kinetic profile of cell growth. Methanol consumption was measured by HPLC analysis and the extracted pigments were analyzed by liquid chromatography/mass spectrometry. Chemical composition and rheological properties of the produced exopolysaccharides were also determined.

**Results and conclusions:**

The best experimental parameters were verified using an initial methanol concentration of 7 g/L in the culture medium. The same initial substrate concentration was used in the fed-batch operation and after 60 h of cultivation 5 g/L of biomass were obtained. The accumulation of carotenoids associated with cell growth was monitored, reaching a concentration of 1.6 mg/L at the end of the process. These pigments were then analyzed and characterized as a set of xanthophylls (oxidized carotenoids). In addition, two other product types were identified during the fed-batch operation: exopolysaccharides, which reached a concentration of 8.9 g/L at the end of the cultivation, and an intracellular granular structure that was detected by transmission electron microscopy (TEM), suggesting the accumulation of polyhydroxyalkanoate (PHA), most likely polyhydroxybutyrate.

**Novelty and scientific contribution:**

*Methylobacterium organophilum* demonstrated a unique ability to produce compounds of commercial interest. The distinct metabolic diversity of this bacterium makes room for its use in biorefineries.

## INTRODUCTION

With alarming population growth, there has been a demand for protein production from alternative sources. In this context, the idea of protein production from unicellular organisms such as bacteria, the so-called single-cell protein, has reemerged. This process can be used for protein supplementation in a staple diet, replacing conventional food sources such as soybean and cattle meat, and animal feed (fishmeal). The production of microbial proteins from waste presents a possibility to reduce the operational costs of protein production and consequently lower the product's final price. Their production would also offset the high costs of conventional feedstock (primary biomass), making food production less land-dependent, and thus reducing the pressure on agriculture (*1*). Several already described microbial species can be used for this purpose (*2*-*5*).

Methylotrophic bacteria are a diverse group of microorganisms with a large number of specialized enzymes that allow them to grow on low carbon substrates without carbon-carbon bonds and use them as energy and carbon sources (*6*, *7*). Methanol is a typical carbon substrate for many methylotrophic bacteria. While subgroups of these bacteria can use methane, other species use methylated sulfur, methylated amines and halogenated hydrocarbons, such as chloromethane, bromomethane and dichloromethane. Phylogenetically, most well-characterized methylotrophic bacteria are subclasses of Proteobacteria. Aerobic methylotrophic bacteria can use a CO_2_-reduced carbon source. Methylotrophic genomes have been sequenced, and significant progress in elucidating the specific metabolism of such bacteria has been made (*8*).

Methanol conversion occurs in several steps with formaldehyde as the central intermediate. It is initiated by methanol dehydrogenase, which oxidizes a C1 (single carbon) substrate to produce formaldehyde. This enzymatic step is catalyzed by a periplasmic enzyme-dependent quinone pyrroloquinoline in Gram-negative bacteria, which channels electrons to the oxidized terminal, or by a phosphorylated form of nicotinamide adenine dinucleotide (NADP)-dependent enzyme in Gram-positive bacteria (*9*). A multitude of ways exist to convert highly toxic intermediate formaldehyde in microbial cells to CO_2_. This metabolic pathway was first discovered in *Pseudomonas extorquens* and is widespread among known methylotrophic bacteria (*10*).

Methanol is one of the building blocks in the chemical industry and can be synthesized from petrochemical or renewable resources such as biogas. Considering that methanol can be produced by the conversion of methane from natural gas, the price of methanol has tended to decrease, making it an attractive substrate in processes involving microorganisms for the production of metabolites that add value to the bioprocess. Whitaker *et al.* (*11*) mentioned that the yield of a product obtained from methanol is similar to that obtained from glucose on a carbon-carbon basis.

Methylotrophic bacterium bioprocessing technology has been studied in recent decades as it has been intended for the large-scale production of single-cell proteins. *Methylobacterium organophilum* is a Gram-negative *Bacillus*-shaped bacterium, denominated PPFM (pink pigmented facultative methylotrophic), and classified as type II according to its membrane arrangement and assimilation of carbon compounds *via* the serine pathway. O’Connor and Hanson (*12*) reported that the main enzymes responsible for bacterial cell growth involved in serine pathway are hydroxypyruvate reductase, serine glyoxylate aminotransferase, methanol dehydrogenase and glycerate kinase. The species *M. organophilum* has been studied for the production of biopolymers, such as polyhydroxybutyrate (PHB), exopolysaccharides (EPS), carotenoids, ectoin and vitamins (*13*-*18*).

Polysaccharides can be part of the microbial cell wall in the form of lipopolysaccharides and they can be attached to the cell wall as capsular polysaccharides or secreted to the extracellular medium in the form of EPS (*19*, *20*). They can form highly viscous aqueous solutions, even at low concentrations, which is considered an attractive feature for food, cosmetic and pharmaceutical industries. EPS can even be used *in natura* as safe food additives (*21*). Bacterial polysaccharides have already been used in the industry (*22*) and they can be compared to the gums produced by other natural sources, such as seaweeds and plants (*23*).

Xanthophyll-type carotenoids are pigments responsible for various colors of many vegetables, flowers and plants in general, and many microorganisms are capable of producing them. Their beneficial effects on health have aroused the interest of the scientific community worldwide. The animal feed, pharmaceutical and cosmetic sectors have invested in the research of these important molecules in order to find new sources and applications. Studies involving carotenoids also look for the methods of maintaining an adequate amount of these substances in different foods so that their properties are not lost during processing and storage (*24*), which reaffirms the application of methylotrophic species as a possible animal feed producer, since they are containing carotenoids that provide additional nutritional value.

Production of biopolymers such as polyhydroxyalkanoates (PHAs) has been associated with methylotrophic bacteria, including *M. organophilum*. PHAs are biodegradable and biocompatible biopolymers that can be produced from renewable sources (*25*). Depending on their composition, their properties vary, with approx. 150 monomers reported in the literature, making this compound suitable for various applications (*26*).

Polyhydroxybutyrate (PHB) is one of these existing homopolymers, with properties similar to polypropylene. PHB can be synthesized inside the cell in the form of granules located in the bacterial cytoplasm, which can occupy a good portion of the cytoplasm without changing the osmotic pressure in the cell. It functions as an energy reserve in excess carbon conditions with limited nutrients (*26*-*28*).

Within this context, this paper exploits the metabolic diversity of *M. organophilum* grown on methanol as the sole carbon source in basic mineral medium. Different operation strategies and their implications in cell metabolism are evaluated, and the potential of this bacterium to produce high value-added molecules is investigated.

## MATERIALS AND METHODS

### Microorganism and medium composition

The bacterium under study, *Methylobacterium organophilum* DSMZ-18172, was acquired from the Deutsche Sammlung von Mikroorganismen und Zellkulturen (DSMZ). The cells were activated with basic mineral media, as described in a related work (*15*) available in the literature, and as suggested by DSMZ for this species. The components of the mineral medium were: KNO_3_ (1 g/L), MgSO_4_·7H_2_O (0.20 g/L), CaCl_2_·2H_2_O (0.02 g/L), Na_2_HPO_4_ (0.23 g/L), NaH_2_PO_4_ (0.07 g/L), FeSO_4_·7H_2_O (1 mg/L), CuSO_4_·5H_2_O (5 µg/L), H_3_BO_3_ (10 µg/L), MnSO_4_·5H_2_O (10 µg/L), ZnSO_4_·7H_2_O (70 µg/L), Na_2_MoO_4_·5H_2_O (10 µg/L) and CoCl_2_·6H_2_O (5 µg/L). After solubilization, the liquid mineral medium was sterilized in an autoclave at 121 °C for 20 min. Methanol as substrate was added in all tests at a later stage after cooling the mineral medium to avoid loss by evaporation during the entire process. Bacteria were added after all necessary elements for cell growth were provided in the flasks.

### Cell production in shake flasks

Bacterial growth time of the preinoculum added to all flasks was 40 h using a methanol concentration of 5.0 g/L. The inoculum was centrifuged at 10 956×*g* for 20 min, the supernatant was removed and the cells were resuspended in a mineral medium. These aliquots were centrifuged, then the supernatant was removed (residual methanol was sent for analysis) and the pellets were resuspended in 2 mL of distilled water for absorbance measurement at 600 nm in a spectrophotometer (UV-1800; Shimadzu, Kyoto, Japan). Growth assays were started by mixing 10 mL of the activated inoculum of the bacterial suspension with 190 mL of mineral medium (*φ*=5%) containing methanol concentrations of 1, 4, 7, 12 and 18 g/L in 500-mL conical flasks. The bacterial cultures were incubated at 30 °C and 4×*g* on a rotary shaker, for 84 h (New Brunswick™ Innova 44®; Eppendorf, Enfield, CT, USA).

### Cell production in a fed-batch operation

A Biostat B bioreactor (B. Braun Biotech International, Melsungen, Germany) with automatic control was used for this assay. Cultivation started with 7 g/L methanol and its consumption was verified by liquid chromatography. The dissolved oxygen was monitored online throughout bacterial cultivation with an electrode connected to the bioreactor. The methanol added to the batch at time intervals (26, 36, 42 and 50 h) was diluted with 75 mL of mineral medium containing *φ*(methanol)=30%. This strategy circumvented the problem of methanol evaporation during its handling (given its significant volatility), providing both the substrate and the essential elements necessary for cell metabolism, maintaining a good carbon to nitrogen ratio, without limiting cell growth and production of other compounds. The fed batch bioreactor conditions were as follows: temperature 30 °C, stirring velocity 450 rpm, pH=7, specific aeration rate 9 L/min and the total bioreactor volume capacity 10 L (considering its headspace), which operated with a working volume of 6 L. This strategy helps to control the foam formed in the medium, due to the combination of aeration and mechanical agitation. The pH was maintained constant by adding solutions of 1 M NaOH or 1 M HCl, automatically in the bioreactor.

### Determination of cell concentration

To monitor cell growth kinetics, the cell concentration was determined gravimetrically using a standard calibration curve obtained by measuring the absorbance at 600 nm for different dry cell mass. From this correlation, the calibration curve used in the tests to estimate the concentration of *Methylobacterium organophylum* was based on the following equation:

y=1.56x+0.03 (R^2^=0.998) /1/

### Methanol quantification

The methanol concentration in the samples was determined by high-performance liquid chromatography (HPLC) equipped with a refractive index detector (Waters 2414; Waters, Milford, MA, USA). The column used was a PL Hi-Plex H8 micron, 300 mm×7.7 mm. Sulfuric acid diluted in distilled water in the concentration of 0.005 mol/L was used as the mobile phase at a flow rate of 0.6 mL/min and a maximum pressure of 0.5 Pa. Methanol P.A. (Vetec Fine Chemicals, Duque de Caxias, Brazil) was used as a standard. The column temperature was approx. 60 °C, and the injection volume was 20 μL. The concentrations of the samples were calculated by comparison with the standard concentration of methanol calibration curve.

### Essential amino acid analyses

The protein content was determined by the Kjeldahl method (*29*) and calculated as a mass fraction (%) in each sample. Ash was determined using sample mineralization in a muffle after 6 h at 550 °C and then cooled in a vacuum desiccator and weighed. Carbohydrate contents were estimated using the DuBois method (*30*). The ash content corresponded to the difference between the mass of the sample in the crucible and the empty crucible (*29*). The carbohydrate concentration was calculated using a standard glucose correlation curve. Amino acid analysis (an isocratic elution was used) was performed by HPLC (Agilent 1260 Infinity HPLC System, Santa Clara, CA, USA) using a reversed-phase C18 column, 250 mm×4 mm, 5 µm, flow 1.0 mL/min (Restek, Böckten, Switzerland) with 0.05 M ammonium acetate (pH=6.8 adjusted with phosphoric acid) as mobile phase A and 0.1 M ammonium acetate (pH=6.8) in acetonitrile/methanol/water (44:10:46) as mobile phase B. Detection occurred by UV at 254 nm with a column oven temperature of 52 °C and an injection volume of 40 µL, with standards of 20 amino acids (alanine, arginine, asparagine, aspartic acid, tyrosine, methionine, glutamic acid, histidine, glycine, isoleucine, leucine, lysine, serine, phenylalanine, proline, glutamine, threonine, tryptophan, OH-proline and valine) (*30*). For the preparation of standards, different masses (between 7 and 210 mg) of each amino acid were weighed in individual 10-mL flasks (tryptophan and OH-proline were dissolved in water and prepared separately). Then 0.1 M hydrochloric acid was added, the flasks were treated with ultrasound (Prolab, São Paulo, Brazil) for approx. 10 min and then shaken to homogenize the solution. The aliquots of 100 µL of each amino acid solution were placed into a small glass vial to make up the standard mixture, and from that 100-µL aliquots of the standard mixture were transferred to a glass container for use in the HPLC analysis.

### Carotenoid extraction, quantification and identification

An aliquot of 2 mL of the bacterial suspension was centrifuged (10 956×*g* for 10 min; Megafuge™; Thermo Fisher Scientific, Cambridge, UK) and the supernatant was discarded, leaving the bacterial biomass as a precipitate. The cells were suspended in methanol (2 mL) and were then broken up by shearing with small-diameter glass beads (0.5−1 mm) in a vortex agitator (K 550-G Genie; Scientific Industries Inc., Bohemia, NY, USA) for 10 min at 1000 rpm. The sample was centrifuged for 15 min at 10 956×*g*, and the supernatant was then collected. This process was repeated several times until the pigment was completely removed. Quantification of the carotenoids using spectrophotometry was done as described by Davies (*31*) and mass fraction of carotenoid (in µg/g) was calculated according to the following equation:

*w*(carotenoid)=*A*∙*V*/*ε*∙*m* /2/

where an absorption coefficient for 1% methanol solution in *d*_cuvette_=1 cm was *ε*=2550 mol/(L·cm) as reported by Davies (*31*), the absorbance (*A*) values measured at 450 nm were obtained from previous sample scans with a spectrophotometer (UV-1800; Shimadzu), *V* was the dilution volume (mL) and *m* was the mass of the dry cells in the sample used in the assay. Eq. 2 was used to calculate the mass of carotenoids per mass of cells, which was related to the concentration in the bioreactor at the end of the fed-batch experiment that led us to determine the carotenoid mass fraction in the final fermentation media. Mass spectrometry analysis was performed on a Nexera X2 ultra-high efficiency liquid chromatography system from Shimadzu coupled to a Max Impact mass spectrometer (Maxis Impact, APCI-Q-TOF configuration; Bruker, Billerica, MA, USA). Chromatographic separations were performed on a Hypersil octadecylsilyl (ODS) column (150 mm×2.1 mm, 3 μm particle size) with Hypersil ODS precolumn (1 mm×2.1 mm, 3 μm+0.22 μm in-line filter; Thermo Fisher Scientific, Jundiaí, São Paulo, Brazil). Water with *φ*(formic acid)=0.1% was used as mobile phase A, and methanol with 0.1% formic acid as mobile phase B. The oven temperature was 40 °C, with an injection volume of 25 µL. The spectrometer was operated in scanning mode in the range of *m/z*=50-1200 with an atmospheric pressure chemical ionization (APCI) source in positive ion mode. The mode was data-dependent acquisition (DDA/AutoMS), with isolation/fragmentation of two precursors per cycle. The mass values ​​of the peaks and their found fragments were compared with databases available on PubChem (*32*).

The extracted pigment was analyzed by liquid chromatography coupled to mass spectrometry, and the results were processed with MZmine 2.53 software (*14*).

### Chemical and rheological analyses of the exopolysaccharides

After exopolysaccharide accumulation (36 h), it was not possible to separate the cells from the polysaccharides using centrifugation only. Thus, to circumvent this problem, ice cold ethanol was added to the samples, which was then stirred continuously in a vortex before centrifugation. Two fractions were obtained: a gelatinous fraction containing the exopolysaccarides (supernatant) and bacterial cells (pinkish colored) which settled down to the bottom of the cuvette. As already described by Vijayendra *et al*. (*21*), cold ethanol can be added and subjected to continuous stirring to separate the EPS. In this procedure, the cells are separated from the EPS after centrifugation to measure their mass. The pink color characteristic of the cells allows you to visually verify this separation between them and the more viscous compound produced by the cell. The supernatant was separated and dried in an oven at 60 °C overnight, and the residual mass (EPS) after acid hydrolysis with 1 M H_2_SO_4_ at 80 °C for 2 h was qualitatively and quantitatively analyzed for carbohydrate identification by liquid chromatography (model 2414; Waters, Milford, MA, USA). A PL Hi-Plex H8 micron 300 mm×7.7 mm column (Waters) was used, and 0.005 mol/L H_2_SO_4_ was used as the mobile phase at a flow rate of 0.6 mL/min and a maximum pressure of 0.5 Pa. Rheological analysis of the EPS was performed in an AR-2000 advanced rheometer with an environmental test chamber (ETC) oven (AR-2000, with ETC furnace; TA Instruments (New Castle, DE, USA) using approx. 3 g dried sample, which was suspended to measure the shear rate and apparent viscosity. Oscillatory and steady-state measurements were performed at room temperature, which allowed determination of the elastic (*G’*), loss modulus (*G*’’) and complex viscosity moduli as a function of angular frequency. In dynamic mode, the moduli of the materials are often measured. One such component is the elastic or storage modulus (*G*’), which is directly proportional to the energy stored in a deformation cycle. This is called the loss or dissipative modulus (*G*’’) and it measures the energy dissipated or lost in the form of heat per deformation cycle. These viscosimetric properties provide essential information about the material's microstructure and its predominant behavior.

### Transmission electron microscopy images

Transmission microscopy was performed with JEOL 1200EX (Tokyo, Japan) microscope operated with an electron acceleration voltage of 80 kV with a Megaview III camera (12 bits) at 2K resolution. In the sample fixation stage, 25% glutaraldehyde in 0.2 M sodium cacodylate buffer was used for one hour at room temperature, followed by resolubilization with 0.1 M sodium cacodylate buffer solution (3 times) and Milli-Q water (both from Sigma-Aldrich, Merck, St. Louis, MO, USA). In the postfixation stage, 1% osmium tetraoxide (OsO_4_) (Sigma-Aldrich) with 0.1 M sodium cacodylate buffer (1:1) was used for 1 h, with subsequent washing, followed by serial dehydration in acetone solutions (30, 50, 70, 90 and 100%; 15 min each). The infiltration step was performed with the Epon epoxy resin (Shell Chemical. Co., Houston, TX, USA) at room temperature. Pure resin was used, leaving it to dry for 24 h. The inclusion was carried out by transferring the sample to silicone molds and then including the Epon resin after removing all bubbles. After that, the samples were placed in an oven at 68 °C for 72 h, and then observed at different wavelengths (500 to 5000 nm). Inclusion is the stiffening of the structure through infiltration of resin. Once the resin has infiltrated, the material is inserted into the molds, allowing extremely thin cuts to facilitate the passage of the electron beam through the material.

### Polyhydroxyalkanoate staining by Sudan black

Sudan black (Vetec Fine Chemicals) was used as a 70% (*V*/*V*) ethanol solution. A sample of the cell culture was smeared on a Petri dish and then dried for fixation onto the plate. Sudan black solution was then dripped all over the plate and left for 20 min. The plate was washed with distilled water to remove the excess Sudan black, and then 90% ethanol was dripped onto the plate, which was left for 5 min, followed by washing with distilled water again until total removal of the used solutions (*25*). Sudan black is a dark brown to black powder with a melting point of 120–124 °C and maximum absorption of the solution between 596–605 nm (*25*).

## RESULTS AND DISCUSSION

### Cultivation of Methylobacterium organophilum cells in shake flasks

**Fig. 1:**
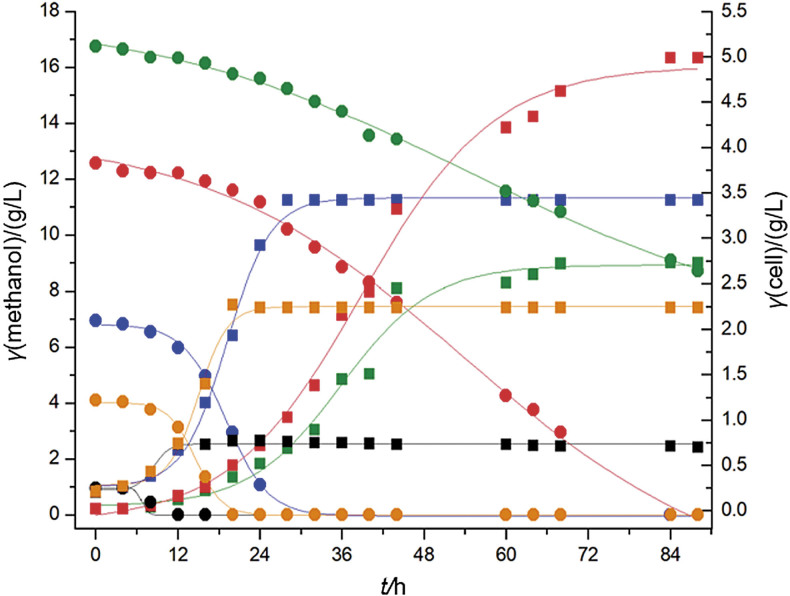
Microbial biomass production (squares with color corresponding to the initial concentration of methanol) under different methanol concentrations (dots): 1 (black), 4 (orange), 7 (blue), 12 (red) and 17 (green) g/L. Experiments were carried out under the following conditions: temperature 30 °C, agitation 250 rpm, pH=7 and methanol consumption rate from 0.10 to 0.25 g/L

To evaluate the ability of the strain to consume methanol as well as the potential inhibitory/toxic effects from its consumption, various initial methanol concentration were assayed. The results in Fig. 1 demonstrate the distinct metabolic machinery of the bacterial strain used in this study regarding its ability to take up methanol at concentrations considered toxic to a large number of living organisms (*33*). The most important point to consider the possibility of methanol oxidation to formaldehyde in methylotrophic bacteria is the presence of methanol dehydrogenase (MDH), an enzyme that contains pyrroloquinoline quinone. The presence of MDH in the bacterial periplasm probably reduces the typical toxicity of methanol when compared to its action in the cells that do not have this enzyme. The high affinity for methanol makes its conversion in the cell faster (*9*).

*M. organophilum* was able to consume methanol at concentrations as high as 17 g/L. At methanol concentrations up to 7 g/L, the bacterium was able to consume all of it with the highest overall uptake rate of 0.25 g/(L·h). Methanol was also completely consumed when its initial concentration was increased to 12 g/L; however, a longer cultivation time was required (84 h). In the medium with the highest tested methanol concentration (17 g/L), only 48% was consumed after 88 h, when the fermentation was interrupted since the bacterial growth had ceased. Although it showed the ability to consume methanol, this bacterium was sensitive to the toxic effects caused by the increasing concentration of alcohol, which was reflected in the reduction of the methanol uptake rate as well as in the specific growth rate (above 7 g/L) and in the cell growth yield, which decreased as the methanol concentration increased (Table 1). The C:N ratios were calculated based on the concentrations of methanol and potassium nitrate since the medium contained only methanol as a carbon source in a solution of mineral salts, with KNO_3_ as the only nitrogen source. The highest cell concentration on cell dry mass basis (approx. 5 g/L) was achieved with an initial methanol concentration of 12 g/L; however, the volumetric productivity of cells was approximately half of that obtained with an initial methanol concentration of 7 g/L. At the latter initial methanol concentration, the highest value of volumetric productivity was 0.115 g/(L·h) and overall substrate consumption rate of 0.250 g/(L·h) was reached. A study carried out by Jafari *et al*. (*33*) using *Methylobacterium* showed that there is an optimum methanol concentration (18 g/L) for cell growth, and above this concentration, methanol inhibits cell growth. Methanol concentrations in the range of 10 to 12 g/L generated higher cell concentrations. With a methanol concentration below this range, there was not enough carbon source to promote bacterial growth and at a methanol concentration above this range (20 g/L or more), the toxic effect of methanol inhibited growth (*33*).

**Table 1:** Growth response variables for different concentrations of methanol in flasks

*γ*(methanol)/ (g/L)	C:N	*X*_max_/ (g/L)	*μ_x_*/h^-1^	Substrate reduction/ %	*t*_SR_/ h	*Y*_X/S_/ (g/g)	*Q*_S_/ (g/(L**^.^**h))	*Q*_X_/ (g/(L**^.^**h))
1	3	0.77± 0.02	0.120±0.007	100	12	0.57±0.01	0.080± 0.001	0.060± 0.007
4	11	2.27± 0.05	0.140± 0.014	100	20	0.52±0.01	0.20± 0.03	0.10± 0.01
7	19	3.42± 0.02	0.100± 0.007	100	28	0.46±0.02	0.250± 0.007	0.115± 0.001
12	31	4.990±0.007	0.080± 0.007	100	84	0.40±0.01	0.140± 0.007	0.06± 0.01
17	45	2.73± 0.02	0.070± 0.007	48	88	0.32±0.02	0.090± 0.008	0.030± 0.007

*X*_max_=maximum cell concentration, *µ*_x_=specific growth rate, *t*_SR_=time for substrate reduction, *Y*_x/s_=cell growth yield, *Q*_S_=overall substrate consumption rate, *Q*_x_=volumetric productivity of cells

Methylotrophic bacteria growing aerobically on single-carbon (C1) substrates produce formaldehyde as a central intermediate, which is toxic to cells through its nonspecific reactivity with proteins and nucleic acids (*34*). A key challenge for these microorganisms is how to maximize the flux through formaldehyde while preventing the intracellular pool of free formaldehyde from accumulating to toxic levels (*35*). Methanol dehydrogenase (MDH) catalyzes the conversion of methanol to formaldehyde *via* an oxidation-reduction reaction that occurs in the periplasm. When formaldehyde crosses the cytoplasmic membrane and enters into the cytoplasm, it reacts with either tetrahydrofolate or tetrahydromethanopterin.

The first reaction occurs spontaneously (nonenzymatically) and enters into the serine pathway, and the second (the long route) has been demonstrated to be catalyzed by a formaldehyde-activating enzyme (*36*). However, the condensation rate and/or affinity of these reactions for formaldehyde are lower than of the oxidation of methanol to formaldehyde by MDH (*37*). Thus, formaldehyde tends to accumulate in the cytoplasm, causing toxicity to the cells. Therefore, there should be a balance between the rate of methanol oxidation and the rate of formaldehyde assimilation to prevent or at least alleviate cell toxicity. The higher the oxidation rate of methanol, the greater the accumulation of formaldehyde, which in turn slows down cell metabolism. Table 1 also shows that as the methanol concentration increases, the C:N ratio also increases, but the cell yield decreases. This very likely occurs because high concentrations of carbon can lead to the accumulation of exopolysaccharides and other macromolecules in methylotrophic cells, such as polyhydroxybutyrate (PHB), as reported by Kim *et al.* (*38*), but which were not measured in the flask experiment of the present work. Additionally, as mentioned before, the highest volumetric productivity was achieved at a methanol concentration of 7 g/L, which corresponded to a C:N ratio of 19. Gowda and Shivakumar (*39*) reported that C:N ratios greater than 20 may be metabolically damaging to the cells, and this was observed in the present work since the cell yield gradually decreased as the C:N ratio increased.

### Cell production in mineral medium in the fed-batch instrumented bioreactor

**Fig. 2:**
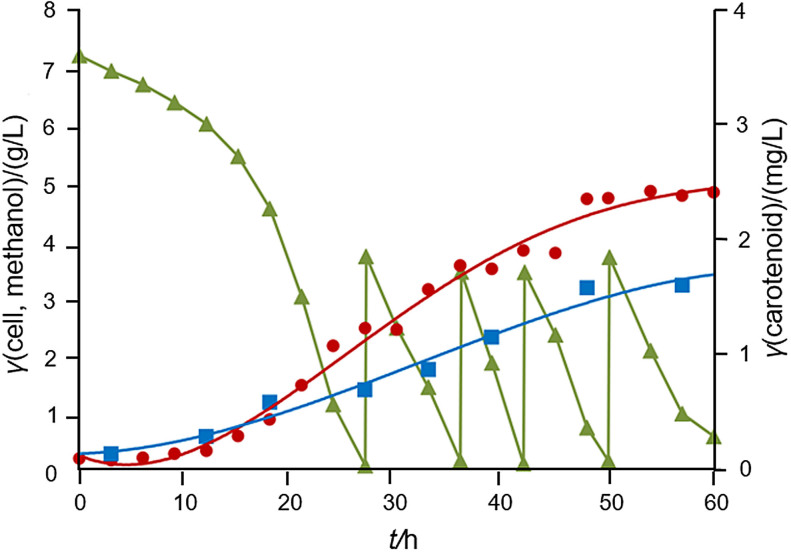
Growth rate of *Methylobacterium organophilum* DSMZ 18172 (dot) and carotenoid production (square) in the fed-batch bioreactor using methanol (triangle) as the sole carbon source. Conditions were as follows: temperature 30 °C, agitation 450 rpm, pH=7, and specific aeration rate 9 L/min

The simple initial batch started with 7 g/L methanol, consumed in approx. 27 h (Fig. 2), when the additional 4.0 g/L of methanol were added to the bioreactor. Measurements of methanol and dissolved oxygen concentrations were used as important parameters to determine the concentration of methanol that needs to be added to the bioreactor culture medium. Methanol and dissolved oxygen concentrations were monitored (by liquid chromatography measurements) to determine the methanol feeding time. This was performed when the methanol was completely consumed and the oxygen amount reached the set point (50% O_2_ saturation), since the cellular metabolic activity ceased when the methanol was depleted in each cycle (Fig. 2). This justifies the decrease in the cell yield obtained in the fed-batch bioreactor. As expected, there was no substrate inhibition during the four feeding cycles, since the methanol was almost totally consumed in all cycles, except in the last one, even though this last cycle resulted in in a high consumption of methanol (85%).

Maintaining the conditions of the fed-batch process for a total of 60 h, at a temperature of 30 °C, stirring at 450 rpm, pH around 7, with the addition of HCl and/or NaOH and at the specific aeration rate of 9 L/min, the maximum cell concentration was 5.05 g/L, maximum specific growth rate (*µ*_max_) 0.11 h^-1^, specific growth rate (*µ*) 0.06 h^-1^, substrate reduction 97.3%, cell growth yield 0.24 g/g, overall substrate consumption rate (*Q*_s_) and volumetric productivity (*Q*_x_) 0.34 and 0.08 g/(L·h) respectively. At the end of the trial, a total of 1.6·10^-3^ g/L of carotenoids was obtained. As shown by Vila *et al*. (*40*), the total carotenoid contents of the isolates were similar, ranging between 0.33 and 0.73 mg/g dry biomass, corroborating the fact that carotenoids are produced in lower amounts.

The production of EPS was confirmed in a strictly mineral medium containing methanol as the sole carbon source. EPS formation possibly occurs due to a deviation in the metabolic pathway related to oxygen limitations in the culture medium, showing a consequent decrease in cell growth. The EPS concentration in the system was estimated from sampling at the end of the process. A 25 mL aliquot was removed from the bioreactor and precipitated with 90% cold ethanol as reported in the previous sections. The cells were separated from the supernatant, dried and weighed on an infrared scale. The dry mass was used to estimate an EPS concentration of 8.9 g/L in the bioreactor.

The hydrolysis of EPS followed by the HPLC analysis showed that the EPS were composed of approx. 50% carbohydrates, with approx. 15% glucose and 35% mannose. The late production (after 36 h of cultivation) of EPS by microorganisms is frequently related to secondary metabolism, often promoting growth during a subsequent shortage (in either of the substrates or other factors), providing some protection or barrier against an inhospitable environment and facilitating cell adherence to solid surfaces (*41*).

**Fig. 3:**
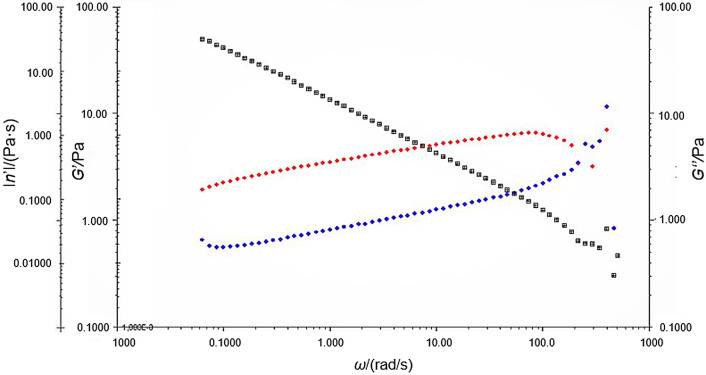
Rheological analysis of exopolysaccharides. *G’*=elastic modulus (red diamond), *G’*’=loss modulus (blue diamond), water as control substance (black diamond), *η’*=viscosity and *ω*=angular frequency

From the EPS rheological analysis, a decrease in viscosity was measured with an increase in the shear rate, suggesting pseudoplastic fluid behavior. Its viscosity was approx. 20 times greater than that of water. The elastic modulus is superior to the viscous modulus, confirming the characteristic of a non-Newtonian pseudoplastic behavior (Fig. 3).

In an analysis by Choi *et al.* (*13*) with *Methylobacterium organophilum* (NCIB 11278 KC-1), the viscosity of the studied biopolymer was 0.018 Pa·s, which is approx. 10 times higher than the viscosity of xanthan gum and approx. 200 times higher than that of pullulan, a polysaccharide excreted by some species of bacteria. The result of this viscometric analysis was similar to that found in this work, which was approx. 0.019 Pa·s.

As demonstrated by Stepnowski *et al.* (*16*), this species (*M. organophilum*) can produce myxol-type pigments. They named the pigment dihydroxylycopene (dihydroxy derivatives of oscillol) or myxol. Based on the known monoisotopic mass of certain carotenoids in general, a search was performed in the MZmine program (*14*), where the mass value was entered to find the *m/z* peaks (load/mass ratio) of each substance. Table 2 shows the *m/z* ratio, retention time and peak intensity of the molecules found in the sample compared to the literature data (*32*).

**Table 2:** Properties of the carotenoids found in the sample, compared with values in the literature (*29*)

	Previous work (*29*)				Present work		
Carotenoid		Standard *m/z*		Molecular formula	*m/z*	*t*_R_/min	Peak intensity
Lutein or zeaxanthin		568.4280	C_40_H_56_O_2_		568.4269	22.29	2.3·10^2^
Myxol		584.4229	C_40_H_56_O_3_		584.4234	22.28	1.5·10^2^
Canthaxanthin		564.3967	C_40_H_52_O_2_		564.3977	24.84	2.0·10^2^
Astaxanthin		596.3866	C_40_H_52_O_4_		596.3871	18.85	1.2·10^2^
Spheroidene		568.4644	C_41_H_60_O		568.4644	24.30	1.6·10^2^
1,1’-dihydroxylycopene		572.4593	C_40_H_60_O_2_		572.4594	21.83	1.6·10^2^

Electrospray ionization mass spectrometry analysis showed six fragments that could be identified by this method, with very high precision of the *m/z* values when compared to previous findings (*32*): lutein or zeaxanthin (these compounds have the same molecular formula and mass but a different structural conformation), myxol, canthaxanthin, astaxanthin, spheroidene and 1,1'-dihydroxylycopene. For example, the 1,1'-dihydroxylycopene pigment found by Stepnowski *et al.* (*16*) with a monoisotopic mass of 572.4593 can be considered to be present in the analysed sample, as the monoisotopic mass value found here (*m/z*=572.4594) was very close to the one found in the previous work. Further studies by high-resolution mass spectrometry and ^1^H NMR analysis are needed to confirm these structures.

### Composition of essential amino acids

The most important components of *M. organophilum* are proteins, carbohydrates and lipids. Compared with yeast cells from breweries (*42*), which can be used, for example, as sources of single-cell proteins, as they have lower protein (39%) and total lipid (0.5%) contents, *M. organophilum* is a good source of single-cell proteins.

*M. organophilum* contains (54.1±2.9) % crude protein, (5.4±1.4) % lipids and (17.3±1.9) % carbohydrates. The content of nitrogen was (9.6±4.4) % and of minerals (7.8±0.2) %. The dry matter was (91.5±0.1) % and an unidentified compound was (5.8±0.2) %.

Due to the presence of essential amino acids in its composition, *M. organophilum* biomass presents the potential for application in animal feed formulations and as nutritional supplement for animals. The amino acids alanine, arginine, aspartic acid, tyrosine, glutamic acid, histidine, glycine, isoleucine, leucine, lysine, serine, phenylalanine, proline, threonine, tryptophan and valine were identified. The amino acids methionine, cysteine and asparagine were probably not identified due to the limitations in the methodology of chromatographic analysis, considering that the masses used as standard were in a range between 7 and 21 mg.

The *Rhodocyclus gelatinosus* biomass obtained from poultry slaughterhouse wastewater contained photosynthetic pigments that are mainly responsible for the purple-red coloring (*43*), among which bacteriochlorophyll and carotenoids of the spheroidene series (spheroidene, hydroxysferoidene and spiriloxanthin) stand out. According to Khan *et al.* (*44*), aspartic acid ((17.82±3.97) %) and leucine ((15.90±2.33) %) were identified in higher amounts and methionine was not present, justifying its use as a possible protein for animal feed. A study with the carotenoid‐producing photosynthetic bacterium *Rhodopseudomonas faecalis* by Patthawaro and Saejung (*45*) revealed that chicken manure was the best substrate for the production of single-cell proteins by submerged fermentation using photosynthetic bacteria compared to pig, cow and buffalo manure. The authors suggested this alternative form of SCP production for animal feed, as well as this strategy for the management of animal waste. In the present study, almost all amino acids were identified, and histidine was found at the highest concentration (Table 3 (*43*-*46*)).

**Table 3:** Amino acid composition of different protein sources

Amino acid	*γ*(amino acid)/%				
a	b	c	d	e
Alanine	1.30±0.07	5.4	6.98	13	1.44
Arginine	4.9±0.3	3.1	-	5	1.30
Aspartic acid	0.5±0.2	8.2	5.74	17.8	3.54
Tyrosine	0.70±0.09	2.9	2.9	5	0.87
Glutamic acid	0.40±0.06	7.2	6.85	4.5	2.14
Histidine	17.0±2.8	1.2	1.83	2	1.15
Glycine	1.30±0.04	2.9	4.18	3	1.42
Isoleucine	13.8±2.1	1.8	3.18	5	1.00
Leucine	1.6±0.2	4.9	6.80	15.9	2.05
Lysine	6.30±0.44	4.0	3.61	2.4	3.36
Serine	0.10±0.04	2.6	2.63	3.1	0.47
Phenylalanine	2.9±0.5	2.7	3.03	2	1.85
Proline	1.20±0.06	4.5	3.27	14	1.54
Threonine	0.50±0.12	2.6	3.52	4.3	1.40
Tryptophan	0.90±0.03	-	1.74	-	-
Valine	0.90±0.05	2.9	4.56	4.5	1.16
Methionine	-	0.5	1.40	-	0.25
Cysteine	-	0.5	0.59	-	-
Asparagine	-	-	-	-	-

a=*Methylobacterium organophilum* (this work), b=five strains of the genus *Methylophilus* (*46*), c=*Rhodocyclus gelatinosus* (*43*), d=*Saccharomyces cerevisiae* (*44*), e=*Rhodopseudomonas faecalis* (*45*)

Digestibility tests of protein preparations produced from single-cell protein (SCP) have previously been carried out, so that this type of supplementation could have greater acceptability on the market and by consumers in general. Works such as those by Zhang *et al.* (*47*) using *Corynebacterium glutamicum* and Jones *et al.* (*48*), showed that the use of this bacterium in pig feed (50% traditional meal and 50% SCP) and for the aquatic cultures feed (replacing up to 52% of fishmeal in salmon farming) does not prevent growth or cause any damage to the nutritional profile of the product, even though it is a preparation from different microbial sources, including microalgae, yeast, other fungi and bacteria. All of these sources exhibit unique advantages and challenges, and are actively being investigated and commercialized.

Tlusty *et al.* (*49*) replaced up to 100 and 55% of the fishmeal in a compound feed for white shrimp and salmon, respectively, and tested their growth and consumer taste preference. In each of these tests, the animals performed equivalently when they were fed diets containing either the SCP from *Methylobacterium extorquens* or a standard aquaculture diet. These promising results, as well as a variety of others that can be found in the literature, increasingly encourage the development of technologies for the cultivation of SCP, mainly using innovative sources such as methanol. In short, the development of a feed obtained from a simple substrate, such as methanol, employing a pigmented bacterium that accumulates carotenoids adds another advantage: the production of biomass rich in protein nutrients and oxycarotenoid pigments, which can be used as a pigmenting ingredient in feed for different animals. The use of a meal with similar formulation characteristics for laying hens, as studied by Ponsano *et al.* (*43*), aimed to improve egg yolks and products containing eggs.

In animals, carotenoids exert various protective functions and can effectively destroy reactive oxygen species (free radicals) and inhibit their formation, functioning as antioxidants. In chickens, carotenoids can prevent encephalomalacia. Carotenoids appear to increase the cytotoxic activity, decrease the growth rate of tumors and promote wound healing (*50*-*52*).

### Electron microscopy images and coloration with Sudan black highlighting the accumulated PHA

**Fig. 4:**
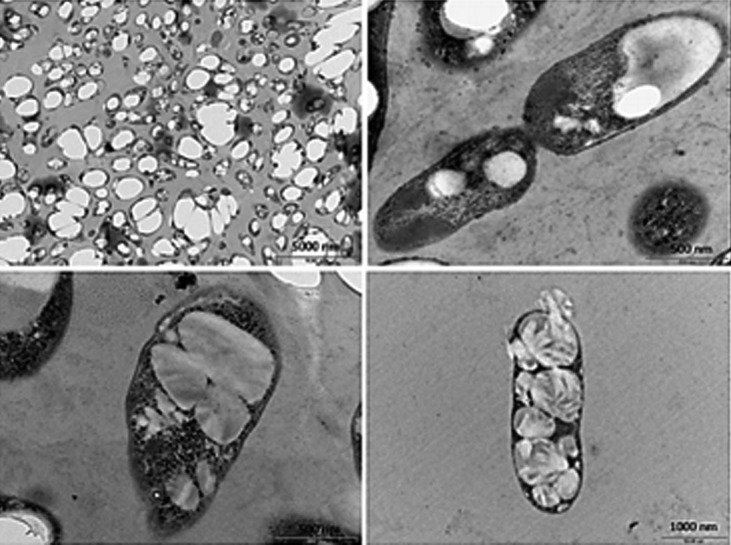
Transmission electron microscopy (TEM) of a sample obtained from the fed-batch assay in bioreactor, showing accumulation of possible PHA granules in the cells of *M. organophilum*

Gowda and Shivakumar (*39*), who studied PHB production, reported that C:N ratios greater than 20 may be metabolically damaging to cells. Increasing the C:N ratio from 10:1 to 20:1 resulted in an increase of 1.6 g/L in cell biomass and caused a large reduction in PHB production (from 72.85 to 2.5%). The authors attribute these results to the rapid use of excess carbon, leading to increased growth due to physiological changes in the cells and disfavor of the production of PHB. This was also observed in the present work, since the cell yield gradually reduced with the increase in the C:N ratio and, consequently, the specific growth rate. The cells were analyzed by a transmission electron microscope, and the images show that granules (probably of PHA) accumulated in the cytoplasm (Fig. 4).

Accumulation in the form of granules seems to distribute between the daughter cells during cell division. These granules are distributed heterogeneously among the cells, where some cells have larger granules than others. The accumulation in the form of a biopolymer shows that excess carbon was an important factor. The granules accumulate as an energy reserve and are protected by a lipid envelope, which can be degraded by the cell if necessary (*25*).

**Fig. S1:**
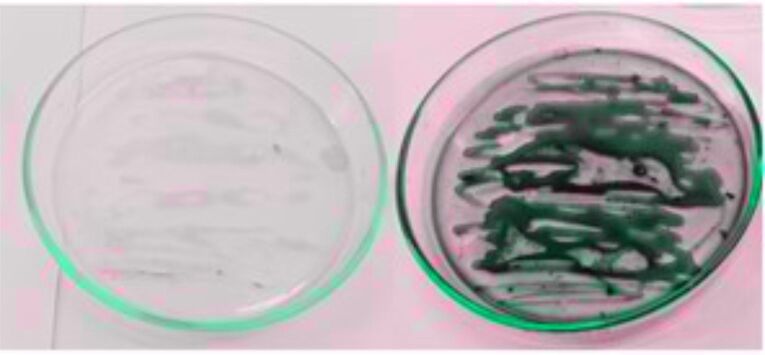
Staining with Sudan Black of *Methylobacterium organophilum* colonies. The first plate represents cells grown in shake flask by simple batch and the second plate shows cells grown in a fed-batch bioreactor

Sudan black is commonly used to confirm the presence of PHA in cells. Some authors relate the chemical affinity of the Sudan black dye to the membranes that surround the intracellular biopolymers, or its use in the signaling of the qualitative accumulation of PHA (*26*-*28*). Sudan black is a lipophilic dye that reacts with phospholipids, neutral fats and steroids in the phospholipid membranes, thus making it possible to contrast the PHB granules. The granules then appear darkish when observed by light microscope and in plate samples (Fig. S1).

## CONCLUSIONS

Methanol, a nonconventional substrate for microorganism growth, has many advantages when it is used as a single-carbon substrate compared to methane, which, as a gas, has reduced solubility in the fermentation medium. Compositional analysis of *Methylobacterium organophilum* cells showed a high percentage of crude protein and the presence of almost all essential amino acids, corroborating the possible use of this microbial biomass as a single-cell protein. Beyond the possibility of producing proteins from this sole carbon source, the production of carotenoids associated with *M. organophilum* growth makes this bioprocess commercially attractive. The biochemical production of carotenoids is very relevant due to the possibility of using a low-cost substrate under simple and controlled cultivation conditions. However, further investigations should be carried out to increase the levels of expression of this important group of provitamins and antioxidants. Finally, there is a possibility of producing exopolysaccharides and polyhydroxyalcanoates, which accumulate through a deviation in the metabolic route, making the bioprocess even more attractive and diverse.
